# Primary Hydatid Cyst of the Gluteal Muscle: A Case Report

**DOI:** 10.7759/cureus.51629

**Published:** 2024-01-03

**Authors:** Oussama El Alaoui, Ousama Jelti, Adnane Lachkar, Najib Abdeljaouad, Hicham Yacoubi

**Affiliations:** 1 Department of Orthopaedics and Traumatology, Centre Hospitalier Universitaire (CHU) Mohammed VI, Oujda, MAR; 2 Department of Traumatology and Orthopedics, Faculty of Medicine and Pharmacy, Mohammed VI University Hospital, Mohammed First University, Oujda, MAR

**Keywords:** surgical margins, pelvic mri, site of infection, gluteal muscles, cyst hydatid

## Abstract

Hydatid disease, also known as cystic echinococcus, is a parasitic infection initiated by Echinococcus granulosus. It primarily affects the lungs and liver, but it can also occur in other organs. Hydatid cysts in the gluteal muscle are an exceedingly rare phenomenon, even in areas with high prevalence. We report the case of a 29-year-old farmer who presented with a painful mass in the gluteal region. The diagnostic findings unveiled the existence of a hydatid cyst within the gluteal muscle managed with complete pericystectomy and chemotherapy with antiparasitic drugs. In regions where hydatid cysts are prevalent, it is essential to include them in the list of potential diagnoses for any cystic mass. Diagnosing such cases can be difficult, and surgery remains the most effective treatment.

## Introduction

Hydatid disease, a zoonosis resulting from infection with Echinococcus granulosus larvae, is prevalent in regions recognized for sheep production. This endemic condition is notably widespread in South America, the Mediterranean, Africa, and the Middle East [1]. Although hydatid disease can manifest in various anatomical sites, instances of cystic echinococcosis occurring primarily and in isolation within muscle tissue are rare [2]. The diagnosis relies on extensive investigation, encompassing meticulous history-taking, clinical evaluation, medical imaging, and serological tests [3]. We present an unusual and exceptional case involving the solitary presence of a hydatid cyst in the gluteal region with no associated hepatic or pulmonary involvement.

## Case presentation

A 29-year-old male, living in a countryside setting with no prior medical history, presented at our university hospital with a gradually enlarging mass in his right gluteal area that had been progressing over the past three years. The patient reported no fever or weight loss during this period.

During the clinical assessment, a rounded, soft mass measuring 11 cm in its largest dimension was observed in the gluteal region. The mass was nonpainful upon palpation, exhibited mobility in both superficial and deep planes, and showed no indications of inflammation. There was no restriction in the mobility of the right hip, and the neurological examination produced normal results.

**Figure 1:**
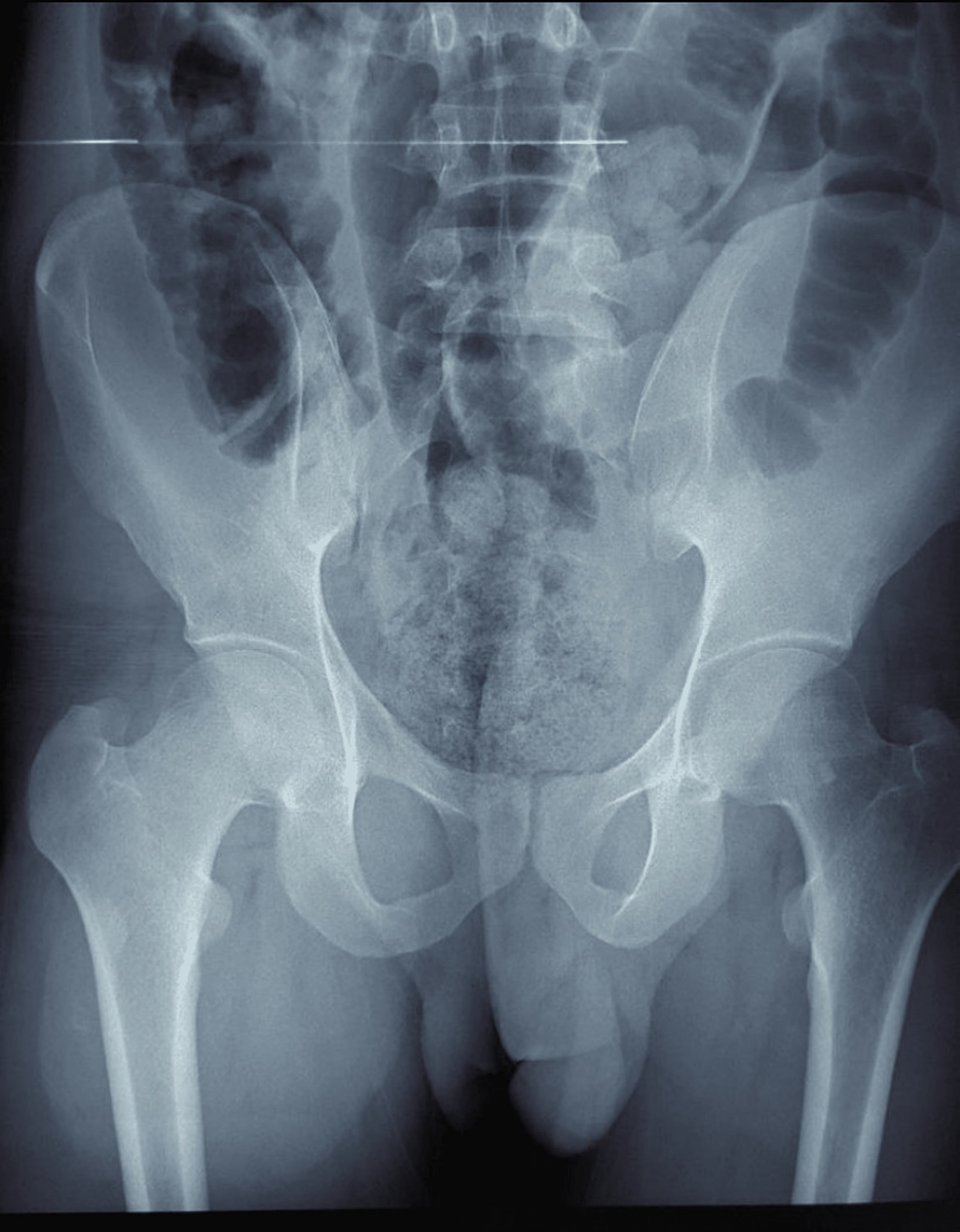
Pelvic X-ray No abnormalities were observed.

Pelvic X-rays were normal, and an ultrasound examination revealed a well-defined cystic lesion with anechoic characteristics (Figure 1).

**Figure 2:**
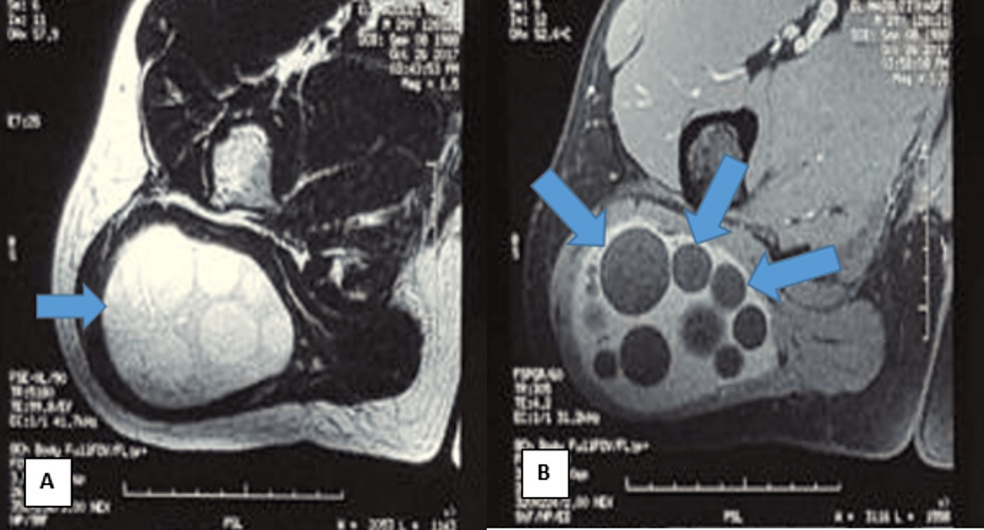
MRI of the pelvis reveals a cystic formation A: Axial T1-weighted imaging displays a cyst infiltrating the right gluteus maximus muscle (blue arrow). B: Axial T2-weighted imaging unveils the appearance of a multivesicular cyst infiltrating the right gluteus maximus muscle (three blue arrows).

MRI assessment demonstrated an oval-shaped formation within the right gluteus maximus muscle, featuring regular contours and hyperintensity on both T1- and T2-weighted imaging. The lesion comprised vesicular structures with a fluid signal, and no enhancement was observed following contrast injection. Measuring 97 x 65 mm and extending approximately 108 mm, this cystic formation induced a deformation of the skin contours in the right gluteal region while respecting the integrity of the femur and other muscular structures (Figure 2).

A computed tomography scan of the thoracoabdominal region showed no other affected areas, but the hydatid serological test came back positive.

**Figure 3:**
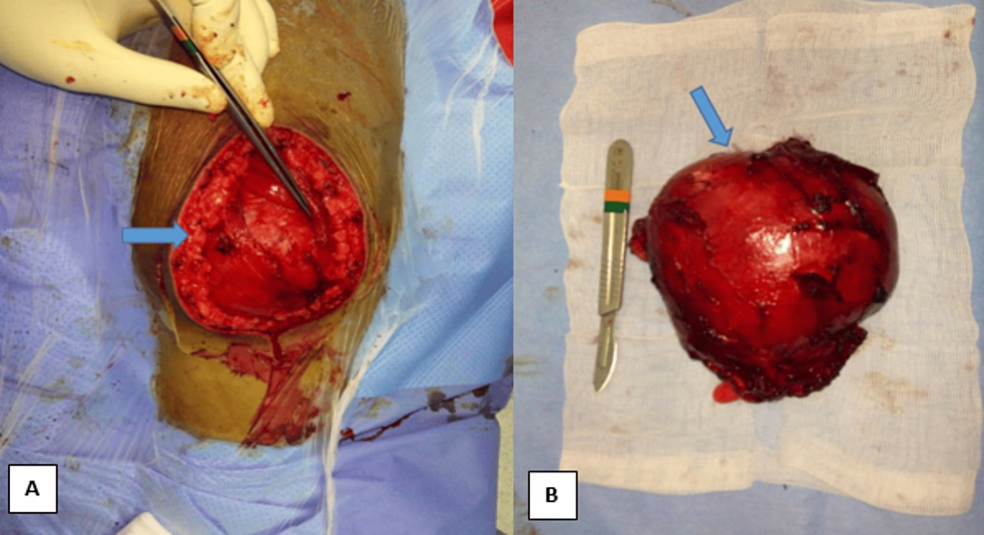
Intraoperative images of the excision of the hydatid cyst A: Operative image depicting the hydatid cyst within the gluteal muscle. B: The macroscopic aspect following the monobloc surgical excision of the hydatid cyst.

The surgical approach was from the back, focusing on the mass, which was found to be a cyst located at the level of the aponeurosis of the gluteus maximus muscle. The surgical team performed a complete pericystectomy without damaging the cyst's integrity (Figure 3).

To protect the surgical field edges, the team used compresses soaked in hypertonic saline solution. After removing the cyst, the surgical specimens were sent for histological examination, confirming the diagnosis of a muscular hydatid cyst. The patient's recovery after the surgery went smoothly, and additional medical treatment with albendazole was started on the third day. The treatment was continued for six weeks after the surgery to prevent any recurrence.

After a follow-up of three years, there was no reappearance of the cyst locally or distantly, and the serological status remained negative.

## Discussion

Echinococcosis is a parasitic infection that impacts both humans and certain mammals. Dogs serve as the definitive host, while sheep are the intermediate host. Humans can assume the role of intermediate hosts through direct contact with an infected dog. [4].

Musculoskeletal hydatid cysts, particularly those in the gluteal region, are extremely uncommon. This rarity can be attributed to the elevated lactic levels in muscle tissue, creating an inhospitable environment for the parasite [4,5].

Typically, these cysts manifest as a persistent, painful mass in the affected area [6]. It is crucial to consider this diagnosis, especially in patients with a history of interaction with dogs and individuals residing in regions where sheep are raised or in rural areas [7]. Serological testing provides a restricted contribution to the diagnostic process because of the increased occurrence of false-negative results [8].

Standard radiography often yields normal results. The primary method of diagnosis still hinges on ultrasound, which demonstrates high sensitivity in typical cases. However, atypical presentations may arise, wherein the lesion may display abnormal characteristics, with or without internal echogenicity [9].

Magnetic resonance imaging is regarded as the most reliable method for evaluating large cysts. It can effectively analyze the cyst's size, interact with surrounding tissues, and influence neurovascular structures [10]. The diagnostic process is facilitated by observing daughter cysts and intracystic membranes, along with identifying a peripheral rim that shows a relatively diminished signal on T2-weighted images [11,12].

The management of muscular hydatid cysts primarily requires surgical intervention. A meticulous surgical approach is crucial to avoid unintended cyst rupture. Although complete excision with total pericystectomy is considered the optimal approach, it may not always be achievable, especially in cases where the cyst is compromised or deeply situated [13,14].

Considering medical treatment with antihelminthic drugs, both before and after surgery, is worthwhile. The goal is to reduce the risk of local recurrence [15].

Innovative approaches have recently surfaced in the treatment of muscular hydatid cysts. According to a study by Necati et al. [16], the management of hydatid cysts incorporated a puncture-aspiration-injection method using alcohol and a sclerosing agent. The outcomes were promising, leading to the conclusion that this technique is associated with straightforward postoperative results.

## Conclusions

Our study underscores the remarkably rare occurrence of the primary echinococcal cyst localized in the gluteal muscle, an exceptional finding even in endemic areas. The preoperative diagnosis poses a challenge because of the intricate clinical presentations. However, including hydatid disease in the initial list of potential diagnoses can significantly contribute to preventing severe complications and minimizing the likelihood of potentially fatal recurrences.
